# Efficacy and safety analysis of TACE combined with lenvatinib and icaritin in unresectable hepatocellular carcinoma with poor performance status

**DOI:** 10.1186/s12885-026-16322-6

**Published:** 2026-06-08

**Authors:** Haohao Lu, Bin Liang, Chuansheng Zheng, Yanyan Cao, Xiangwen Xia

**Affiliations:** 1 Department of Radiology, Union Hospital, Tongji Medical College, Huazhong University of Science and Technology, Jiefang Avenue #1277, Wuhan, 430022 China; 2 Hubei Province Key Laboratory of Molecular Imaging, Wuhan, 430022 China; 3 Hubei Provincial Clinical Research Center for Precision Radiology & Interventional Medicine, Wuhan, 430022 China

**Keywords:** Hepatocellular carcinoma, Physical condition, Unresectable, ECOG score, Interventional therapy, Transarterial chemoembolization, Targeted therapy, Immunotherapy, Icaritin

## Abstract

**Objective:**

To investigate the efficacy and safety of transarterial chemoembolization (TACE) combined with lenvatinib and icaritin in the treatment of patients with unresectable hepatocellular carcinoma (uHCC) with poor physical condition, aiming to provide a safer and more effective treatment strategy for patients with advanced uHCC and poor physical status.

**Materials and methods:**

Clinical data from 49 patients with uHCC and poor physical condition who received treatment between June 2022 and December 2023 were collected. The patients were divided into two groups based on their treatment method: TACE + LEN+Icaritin group (*N* = 21) and TACE group (*N* = 28). The primary endpoints were the objective response rate (ORR), disease control rate (DCR), and progression-free survival (PFS) of the tumor in both groups. The secondary endpoint was the incidence of treatment-related adverse events (AEs) in both groups.

**Results:**

The ORR and DCR of the TACE + LEN+Icaritin group were significantly higher than those of the TACE group (66.7% vs. 28.6%, *P* = 0.011; 85.7% vs. 53.6%, *P* = 0.030). The mPFS of the TACE + LEN+Icaritin group was longer than that of the TACE group (8.6 months vs. 4.1 months, HR = 0.316, 95%CI: 0.166–0.599; *P* < 0.001). The incidence of hand-foot syndrome (all grades) was higher in the TACE + LEN+Icaritin group compared to the TACE group (*P* = 0.004), but there was no significant difference in the incidence of grade 3/4 hand-foot syndrome between the two groups (*P* = 0.429). The incidence of fatigue and anorexia (all grades) was lower in the TACE + LEN+Icaritin group compared to the TACE group (*P* < 0.05). A total of 66.7% of patients in the TACE + LEN+Icaritin group showed improvement in physical condition after treatment, which was significantly higher than in the TACE group (*P* = 0.022).

**Conclusion:**

TACE combined with lenvatinib and icaritin demonstrates good efficacy and safety in the treatment of patients with uHCC and poor physical condition (ECOG = 2). This combination therapy may be a viable option for improving prognosis and quality of life in patients with uHCC and poor physical condition.

**Supplementary Information:**

The online version contains supplementary material available at 10.1186/s12885-026-16322-6.

## Introduction

Hepatocellular carcinoma (HCC) represents one of the most common abdominal malignancies worldwide and constitutes the predominant pathological subtype of primary liver cancer [1], accounting for approximately 75–85% of cases [2]. According to World Health Organization(WHO) data as of 2020, HCC ranks as the sixth most frequently diagnosed cancer and the third leading cause of cancer-related deaths globally [3]. Owing to the lack of characteristic clinical manifestations in its early stages, the majority of patients become symptomatic only when the disease has progressed to an intermediate or advanced stage [4]. At this point, many patients are no longer candidates for surgical resection due to factors such as underlying liver disease, hepatic functional impairment, high tumor burden, portal vein tumor thrombosis, distant metastasis, and poor general health status [5]. Studies report that fewer than 30% of HCC patients qualify for curative surgical resection [6]. Many individuals with HCC experience symptoms including pain, loss of appetite, and malnutrition [7], which often contribute to a decline in performance status. The Eastern Cooperative Oncology Group (ECOG) performance score is commonly used for such assessments [8]. Several HCC staging guidelines incorporate performance status as a key determinant [9, 10]. It directly influences both tumor staging and therapeutic decisions [11]. For instance, within the widely adopted Barcelona Clinic Liver Cancer (BCLC) staging system, patients with an ECOG score of 1–2 are classified as BCLC stage C, irrespective of tumor size, multiplicity, vascular invasion, or metastatic spread [12]. The BCLC guidelines recommend systemic therapy for stage C patients [13]. However, clinical practice suggests that some patients classified as BCLC C—particularly those upstaged solely due to an ECOG score > 1 in the absence of portal vein tumor thrombus or distant metastasis—may still derive benefit from transarterial chemoembolization (TACE) [14].TACE has become a widely utilized modality for patients with unresectable and advanced HCC [15]. Shuguang Ju et al. [16] reported on 110 BCLC stage C HCC patients treated with TACE combined with apatinib, demonstrating that this regimen is both safe and effective.

Commonly used targeted agents for HCC include sorafenib, lenvatinib, donafenib, apatinib, and regorafenib [17]. Lenvatinib, in particular, is recommended as a first-line systemic treatment for HCC in multiple international guidelines [18]. Its approval in 2017 was based on the phase III REFLECT trial, which demonstrated non-inferiority to sorafenib [19]. Several studies have reported that lenvatinib is associated with a relatively low incidence of treatment-related adverse events (AEs) and is generally well-tolerated [20]. Common AEs include hypertension, fatigue, hand-foot skin reaction, and diarrhea [21]. However, in patients with poor baseline performance status, the combination of lenvatinib with TACE may potentially exacerbate drug-related toxicities, further compromising performance status. This decline may lead to discontinuation of anticancer therapy, reduced patient confidence and treatment adherence, and ultimately adversely impact overall treatment outcomes [22]. Icaritin is a flavonoid compound derived from plants of the genus Epimedium (a traditional Chinese medicine). It is a metabolite of icariin produced via microbial metabolism in the gastrointestinal tract and exhibits diverse biological and pharmacological activities [23]. Icaritin has been shown to modulate immune responses, suppress inflammation, protect neural and cardiovascular systems, prevent osteoporosis, inhibit tumor cell proliferation, and improve performance status [24]. In vitro and in vivo studies indicate that icaritin can inhibit proliferation, reduce viability, and induce apoptosis in various cancer cell lines, including those from liver, prostate, lung, colon, breast cancers, and multiple myeloma [25].

This study aims to retrospectively analyze clinical data from our institution to evaluate the efficacy and safety of TACE combined with lenvatinib and icaritin in the treatment of unresectable HCC patients with poor performance status. Our objective is to propose a safer and more effective therapeutic strategy for this challenging patient population with advanced HCC.

## Materials and methods

### General information

Clinical data were collected from 49 patients with unresectable hepatocellular carcinoma (uHCC) and poor performance status who were treated at the Union Hospital, Tongji Medical College, Huazhong University of Science and Technology from June 2022 to December 2023. Inclusion Criteria: (1) Histologically or radiographically confirmed diagnosis of HCC [26].(2) Multidisciplinary assessment determined the disease as unresectable.(3) Age between 18 and 75 years.(4) Liver function classified as Child-Pugh A-B and performance status (ECOG) score of 2.(5) History of liver cirrhosis.(6) No prior treatment with radiation therapy, chemotherapy, targeted therapy, or immunotherapy. Exclusion Criteria: (1) Tumor thrombus involving both main branches of the portal vein or the portal vein trunk with limited collateral circulation.(2) Severe abnormalities in cardiac, pulmonary, renal, hematologic, neurological systems, or coagulation function.(3) Presence of primary or secondary malignancies in other sites.(4) Allergy to iodinated contrast agents, chemotherapy drugs, lenvatinib, or icaritin.(5) Estimated survival time < 3 months.(6) Incomplete clinical follow-up data. Based on the treatment modalities, the patients were divided into two groups: TACE + LEN+Icaritin group (*N* = 21) and TACE group (*N* = 28). Baseline data collected from both groups included: gender, age, cause of liver cirrhosis, preoperative liver function Child-Pugh classification, AFP levels, number of tumors, maximum tumor diameter, portal vein tumor thrombus, history of hypertension, history of diabetes, total bilirubin, ALT, AST, white blood cell count(WBC), red blood cell count(RBC), and platelet count(PLT).

### Methods

#### TACE [27]

TACE was performed via the femoral artery approach. During the procedure, celiac trunk and superior mesenteric artery angiography were conducted. If preoperative imaging suggested the presence of aberrant tumor-feeding arteries, corresponding angiography was performed. After identifying the tumor’s feeding arteries, a microcatheter was used to perform superselective cannulation of the artery. Conventional TACE was performed, initially injecting a lipiodol + epirubicin emulsion, followed by gelatin sponge particles(350–560 μm in diameter; Alicon Pharmaceutical, Hangzhou, China), until blood flow in the tumor-feeding artery was stagnant. The dosage of epirubicin ranges from 20 mg to 40 mg, and the dosage of iodized oil ranges from 5 mL to 15 mL. The TACE procedure was performed by an interventional radiologist with over ten years of extensive operative experience.

#### Lenvatinib treatment

Dosage: For patients weighing < 60 kg, 8 mg orally once daily; for patients weighing ≥ 60 kg, 12 mg orally once daily. In patients experiencing intolerable adverse reactions during lenvatinib treatment, a dose reduction should be implemented. Lenvatinib was temporarily withheld for one day prior to and three days following each TACE procedure.

#### Icaritin treatment

Dosage: 2.4 g orally, twice daily. Icaritin administration was maintained continuously during the TACE treatment period. No dose reductions, interruptions, or permanent discontinuations of icaritin were required due to drug-related adverse events in this study cohort.

The patient underwent follow-up every 4 weeks, including blood tests, contrast-enhanced CT /MRI, and assessments of general condition and physical status. If the examination confirmed the presence of active tumor lesions, the patient received repeat TACE treatment. The patient was treated with regorafenib and/or tislelizumab following disease progression.

### Observation indicators

The primary study endpoints were: (1) Evaluation of tumor response in both groups post-treatment using the mRECIST criteria, and the comparison of objective response rate (ORR) and disease control rate (DCR) between the two groups; (2) Progression-free survival (PFS) between the two groups. The secondary study endpoints were: (1) Changes in physical status and blood markers in both groups 3 months after treatment; (2) Assessment of treatment-related adverse events in both groups using version 5.0 of the Common Terminology Criteria for Adverse Events (CTCAE).

### Statistical methods

Statistical analysis was performed using SPSS 27.0 software (IBM, Armonk, NY, USA). The normality of distribution for continuous variables was assessed using the Shapiro-Wilk test. For variables meeting normality and homogeneity of variance assumptions, comparisons were made using independent samples t-tests, and results are expressed as mean ± standard deviation. For non-normally distributed variables or those with unequal variances, the Mann-Whitney U test was used, with results expressed as median (interquartile range). Categorical variables are expressed as frequencies (percentages) and were compared using the Chi-square test or Fisher’s exact test, as appropriate. No clinically significant missing data were present for the analyzed variables in the final cohort, as this was an inclusion criterion. Therefore, no imputation methods were applied.

## Results

### Comparison of baseline characteristics between the two groups

There were no significant statistical differences between the two groups in terms of gender, age, etiology of cirrhosis, preoperative liver function (Child-Pugh classification), AFP levels, number of tumors, maximum tumor diameter, portal vein tumor thrombus, history of hypertension, history of diabetes, total bilirubin, ALT, AST, WBC, RBC, and PLT (*P* > 0.05, Table 1).

**Table 1:** Comparison of baseline characteristics between the two groups

			Group				
			TACE + LEN+Icaritin group(*N* = 21)	TACE group(*N* = 28)	Chi-Square Tests(p-value)		t-test(p-value)
					Fisher’s Exact Test	Pearson Chi-Square	
Gender	Female	Count(%)	5(23.8%)	9(32.1%)	0.750		
	Male	Count(%)	16(76.2%)	19(67.9%)			
Etiology of cirrhosis	Hepatitis B	Count(%)	16(76.2%)	20(71.4%)		0.929	
	Hepatitis C	Count(%)	3(14.3%)	5(17.9%)			
	others	Count(%)	2(9.5%)	3(10.7%)			
Pre-treatment liver function	Child A	Count(%)	14(66.7%)	20(71.4%)	0.762		
	Child B	Count(%)	7(33.3%)	8(28.6%)			
Number of tumors	Solitary	Count(%)	13(61.9%)	15(53.6%)	0.771		
	Multiple	Count(%)	8(38.1%)	13(46.4%)			
Maximum tumor diameter	< 5 cm	Count(%)	4(19.0%)	8(28.6%)		0.743	
	5–10 cm	Count(%)	10(47.6%)	12(42.9)			
	> 10 cm	Count(%)	7(33.3%)	8(28.6%)			
Portal vein tumor thrombus	No	Count(%)	12(57.1%)	18(64.3%)	0.768		
	Yes	Count(%)	9(42.9%)	10(35.7%)			
AFP	< 400ug/L	Count(%)	5(23.8%)	2(7.1%)	0.122		
	≥ 400ug/L	Count(%)	16(76.2%)	26(92.9%)			
Hypertension	No	Count(%)	15(71.4%)	23(82.1%)	0.494		
	Yes	Count(%)	6(28.6%)	5(17.9%)			
Diabetes	No	Count(%)	17(81.0%)	21(75.0%)	0.737		
	Yes	Count(%)	4(19.0%)	7(25.0%)			
Age(Years)	Mean ± SD		57.7 ± 12.5	56.8 ± 12.3			0.809
Pre-treatment bilirubin(µmol/L)	Mean ± SD		11.83 ± 5.86	13.11 ± 4.03			0.367
Pretreatment ALT(U/L)	Mean ± SD		50.9 ± 28.3	46.1 ± 24.9			0.533
Pretreatment AST(U/L)	Mean ± SD		29.7 ± 13.3	28.0 ± 10.2			0.628
Pretreatment WBC(G/L)	Mean ± SD		3.25 ± 1.12	3.57 ± 0.88			0.273
Pretreatment RBC(T/L)	Mean ± SD		3.78 ± 0.69	3.95 ± 0.78			0.447
Pretreatment PLT(G/L)	Mean ± SD		94.1 ± 39.6	104.3 ± 37.4			0.362

### Comparison of blood indicators between the two groups three months after treatment

There were no significant differences between the two groups in total bilirubin, ALT, AST, WBC, RBC, and PLT after treatment (*P* > 0.05, Table 2).

**Table 2:** Comparison of blood parameters between the two groups after three months of treatment

		Group		t-test(*p*-value)
		TACE + LEN+Icaritin group(*N* = 21)	TACE group(*N* = 28)	
Post-treatment bilirubin(µmol/L)	Mean ± SD	18.47 ± 8.32	22.88 ± 11.74	0.150
Post-treatment ALT(U/L)	Mean ± SD	51.4 ± 17.7	47.1 ± 20.2	0.439
Post-treatment AST(U/L)	Mean ± SD	44.4 ± 29.9	50.3 ± 26.5	0.470
Post-Treatment WBC(G/L)	Mean ± SD	3.40 ± 0.91	3.08 ± 0.83	0.212
Post-Treatment RBC(T/L)	Mean ± SD	3.84 ± 0.84	3.68 ± 0.70	0.478
Post-Treatment PLT(G/L)	Mean ± SD	87.9 ± 26.8	93.1 ± 28.2	0.513

### Tumor efficacy evaluation three months after treatment in the two groups

The proportion of patients who achieved Complete Response (CR) or Partial Response (PR) in the TACE + LEN+Icaritin group was higher than in the TACE group, while the proportion of patients with Stable Disease (SD) or Progressive Disease (PD) was lower (*P* = 0.036, Table 3). The ORR in the TACE + LEN+Icaritin group was 66.7%, which was higher than the TACE group’s 28.6% (*P* = 0.011, Table 3). The DCR in the TACE + LEN+Icaritin group was 85.7%, significantly higher than the TACE group’s 53.6% (*P* = 0.030, Table 3).

**Table 3:** Tumor efficacy assessment in both groups at three months

			Group		Chi-Square Tests(*p*-value)	
			TACE + LEN+Icaritin group(*N* = 21)	TACE group(*N* = 28)	Pearson Chi-Square	Fisher’s Exact Test
Tumor response	CR	Count(%)	4(19.0%)	1(3.6%)	0.036	
	PR	Count(%)	10(47.6%)	7(25.0%)		
	SD	Count(%)	4(19.0%)	7(25.0%)		
	PD	Count(%)	3(14.3%)	13(46.4%)		
ORR		Count(%)	14(66.7%)	8(28.6%)		0.011
DCR		Count(%)	18(85.7%)	15(53.6%)		0.030

### Comparison of PFS between the two groups

**Fig. 1:**
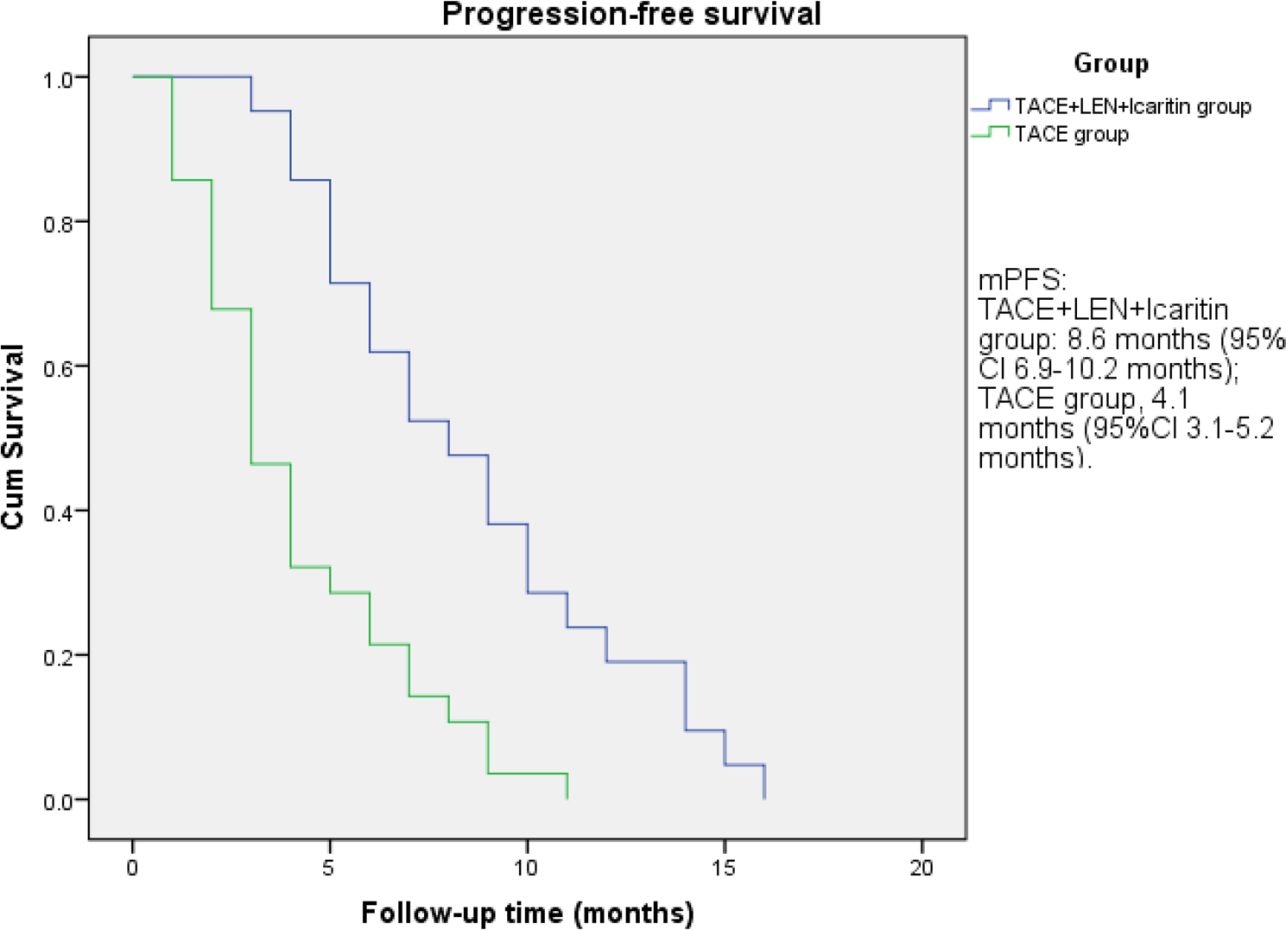
Progression-Free Survival (PFS) of uHCC Patients with Poor Performance Status (ECOG 2) Treated with TACE + LEN+Icaritin vs. TACE Alone. mPFS: TACE + LEN+Icaritin group, 8.6 months (95%CI 6.9–10.2 months); TACE group, 4.1 months (95%CI 3.1–5.2 months)

The mPFS in the TACE + LEN+Icaritin group was longer than that in the TACE group (8.6 months vs. 4.1 months), with a statistically significant difference (HR = 0.316, 95%CI: 0.166–0.599; *P* < 0.001, Table 4; Fig. 1).

**Table 4:** Comparison of PFS between the two groups

Gruop		Median(months)	95% Confidence Interval		Log Rank (Mantel-Cox) (p-value)
			Lower Bound	Upper Bound	
PFS	TACE + LEN+Icaritin group	8.6	6.9	10.2	< 0.001
	TACE group	4.1	3.1	5.2	

### Incidence of treatment-related AEs in the two groups

There were no significant differences in the incidence of abdominal pain, fever, vomiting, hypertension, or diarrhea between the two groups (*P* > 0.05, Table 5). The incidence of hand-foot syndrome (all grades) was higher in the TACE + LEN+Icaritin group compared to the TACE group (28.6% vs. 0.0%, *P* = 0.004, Table 5), but there was no significant difference in the incidence of hand-foot syndrome(grade 3/4) between the two groups (4.8% vs. 0.0%, *P* = 0.429). The incidence of fatigue and anorexia (all grades) was lower in the TACE + LEN+Icaritin group compared to the TACE group (33.3% vs. 67.9%, 23.8% vs. 57.1%, *P* < 0.05, Table 5).

**Table 5:** Incidence of AEs after treatment in both groups

	Group				Chi-Square Tests(*p*-value)	
Adverse events	TACE + LEN+Icaritin group(*N* = 21)		TACE group(*N* = 28)			
	All grades, *n* (%)	Grade 3/4, *n* (%)	All grades, *n* (%)	Grade 3/4, *n* (%)	All grades	Grade 3/4
Abdominal pain	10(47.6%)	4(19.0%)	17(60.7%)	6(21.4%)	0.398	1.000
Fever	7(33.3%)	3(14.3%)	11(39.3%)	6(21.4%)	0.769	0.714
Vomiting	7(33.3%)	1(4.8%)	8(28.6%)	2(7.1%)	0.762	1.000
Elevated blood pressure	5(23.8%)	1(4.8%)	2(7.1%)	0(0.0%)	0.122	0.429
Diarrhea	4(19.0%)	1(4.8%)	1(3.6%)	0(0.0%)	0.150	0.429
Hand-foot syndrome	6(28.6%)	1(4.8%)	0(0.0%)	0(0.0%)	0.004	0.429
Fatigue	7(33.3%)	1(4.8%)	19(67.9%)	7(25.0%)	0.022	0.115
Anorexia	5(23.8%)	1(4.8%)	16(57.1%)	6(21.4%)	0.024	0.214

Note: Hypertension was defined as elevated blood pressure occurring after treatment in patients who either had no prior history of hypertension or had well-controlled hypertension before treatment.

### Improvement in ECOG scores and physical status after treatment in the two groups

The proportion of patients with an ECOG score < 2 in the TACE + LEN+Icaritin group was significantly higher than in the TACE group (*P* = 0.035, Table 6). In the TACE + LEN+Icaritin group, 66.7% of patients showed an improvement in physical status, which was significantly higher than in the TACE group (*P* = 0.022, Table 6).

**Table 6:** Comparison of ECOG scores and improvement in physical condition between the two groups

			Group		Chi-Square Tests(p-value)	
			TACE + LEN+Icaritin group(*N* = 21)	TACE group(*N* = 28)	Pearson Chi-Square	Fisher’s Exact Test
ECOG score after treatment	0	Count(%)	4(19.0%)	1(3.6%)	0.035	
	1	Count(%)	10(47.6%)	8(28.6%)		
	2	Count(%)	7(33.3%)	19(67.9%)		
Physical condition improved	No	Count(%)	7(33.3%)	19(67.9%)		0.022
	Yes	Count(%)	14(66.7%)	9(32.1%)		

### Subgroup analyses for treatment heterogeneity

Key Subgroups Evaluated: Tumor burden, Liver function, Baseline AFP level, PVTT.

(1) Consistent trend of PFS benefit: The combination regimen showed a hazard ratio (HR) of less than 1.0 favoring improved PFS across all predefined subgroups, indicating a consistent trend towards benefit. Formal tests for interaction showed no statistically significant heterogeneity (*P* > 0.05 for all interaction tests), suggesting the treatment effect was generally consistent across different patient characteristics. It should be noted that in the smaller subgroups of patients with tumors < 5 cm (*P* = 0.087, Table 7) and Child-Pugh B liver function (*P* = 0.128,), the P-values did not cross the 0.05 threshold, likely reflecting limited sample size in these strata. Table 7(2) Notable trends: Patients with solitary tumors (HR = 0.28, 95% CI: 0.12–0.63, Table 7) and Child-Pugh A (HR = 0.30, 95% CI: 0.14–0.65, Table 7) exhibited the most pronounced PFS improvements. Patients with PVTT still derived significant benefit (HR = 0.41, 95% CI: 0.18–0.93, Table 7).

**Table 7:** Subgroup analysis of Progression-Free Survival (PFS)

Subgroup	Patients (*n*)	Hazard Ratio (HR)	95% CI	*P*-value	Interaction *P*-value
All patients	49	0.316	0.166–0.599	< 0.001	-
Tumor number					0.112
Solitary	28	0.28	0.12–0.63	0.002	
Multiple	21	0.39	0.17–0.89	0.026	
Max tumor diameter					0.285
<5 cm	12	0.41	0.15–1.14	0.087	
5–10 cm	22	0.29	0.13–0.65	0.003	
>10 cm	15	0.33	0.12–0.90	0.031	
Child-Pugh class					0.064
A	34	0.30	0.14–0.65	0.002	
B	15	0.42	0.14–1.28	0.128	
PVTT status					0.352
Absent	30	0.28	0.13–0.61	0.001	
Present	19	0.41	0.18–0.93	0.034	
Baseline AFP					0.214
<400 µg/L	7	0.35	0.08–1.55	0.168	
≥400 µg/L	42	0.32	0.16–0.63	0.001	

### Multivariable analysis of prognostic factors

1. Independent predictors of improved PFS:
  Combination therapy (HR = 0.35, 95% CI: 0.18–0.68; *P* = 0.002, Table 8).
  Solitary tumor (HR = 0.48, 95% CI: 0.26–0.89; *P* = 0.020, Table 8).
  Child-Pugh A (HR = 0.52, 95% CI: 0.28–0.96; *P* = 0.037, Table 8).
  The multivariable Cox model included 37 progression events.
2. Non-significant factors:
  Age (*P* = 0.32), AFP (*P* = 0.18), PVTT (*P* = 0.11), and tumor diameter (*P* = 0.25, Table 8).

**Table 8:** Multivariable Cox Regression Analysis for PFS

Prognostic Factor	Category	HR	95% CI	*P*-value
Treatment group	TACE + LEN + Icaritin vs. TACE alone	0.35	0.18–0.68	0.002
Tumor burden	Solitary tumor vs. Multiple	0.48	0.26–0.89	0.020
Liver function	Child-Pugh A vs. B	0.52	0.28–0.96	0.037
PVTT status	Absent vs. Present	0.61	0.33–1.13	0.112
Max tumor diameter	< 5 cm vs. 5–10 cm vs. >10 cm	1.05	0.96–1.15	0.253
Baseline AFP	≥ 400 vs. <400 µg/L	1.32	0.87-2.00	0.184
Age		1.01	0.98–1.04	0.318

## Discussion

HCC represents the predominant pathological subtype of primary liver cancer, the patient’s performance status directly influences disease staging, which in turn dictates treatment strategies and prognosis [28]. According to the widely adopted BCLC staging system, patients with an ECOG PS score of 1–2 are classified as BCLC stage C, irrespective of tumor burden or extent [29]. Consequently, considerable heterogeneity in tumor characteristics exists among patients within the BCLC C category [30]. Systemic therapy is recommended for BCLC stage C patients. The design of the BCLC system implies that patients categorized as stage C solely based on a PS of 1 or 2 may still have potentially treatable tumor burdens, and their eligibility for active antitumor therapy should not be summarily dismissed. Several investigators have explored aggressive treatments in select BCLC C patients, demonstrating survival benefits—particularly in those with limited tumor burden and absence of main portal vein invasion or distant metastasis [31]. Thomas Yau et al. [32] conducted a study involving 3,856 HCC patients, which revealed that within the Hong Kong Liver Cancer (HKLC)-II subgroup classified as BCLC-C, radical treatment provided significantly greater survival benefits compared to systemic therapy (5-year survival rate: 48.6% vs. 0.0%; *P* < 0.0001). TACE exerts its antitumor effects through dual mechanisms: intra-arterial delivery of chemotherapeutic agents directly to the tumor bed induces apoptosis and inhibits proliferation, while subsequent embolization of the tumor-feeding arteries induces ischemic necrosis through hypoxia [33]. In this study, the observed ORR, DCR, and mPFS in the TACE group were 28.6%, 53.6%, and 4.1 months, respectively, consistent with previously published data. Yan Zhao et al. [34] analyzed 15 studies on TACE in advanced HCC and concluded that TACE can be safely administered to advanced HCC patients with vascular invasion or extrahepatic spread and may improve overall survival. According to WHO criteria, the average ORR for TACE in advanced HCC patients is approximately 35% (reported range: 16–61%) [35].

However, the ischemia and hypoxia induced by TACE can upregulate hypoxia-inducible factor-1α (HIF-1α), which in turn increases the levels of VEGF, FGF, and other factors, potentially leading to tumor recurrence and metastasis [36]. To enhance the efficacy of TACE, many researchers have explored combined treatment strategies [37]. One common approach is the combination of TACE with targeted therapy [38]. Molecular targeted drugs frequently used in HCC include sorafenib, lenvatinib, donafenib, apatinib, and regorafenib [39]. These tyrosine kinase inhibitors (TKIs) inhibit multiple receptor tyrosine kinases such as VEGFR, PDGFR, and FGFR, as well as various Raf kinases, thereby suppressing tumor cell proliferation and angiogenesis. This mechanism allows TKIs to synergize with TACE [40]. Lenvatinib, which targets VEGFR, PDGFR, and FGFR, has demonstrated synergistic effects when combined with TACE [41]. This has been confirmed in clinical practice, and in this study, we also used TACE in combination with lenvatinib. Zhenwei Peng et al. [42] reported a study in which 338 patients were randomized across 12 centers in China: 170 patients received LEN-TACE and 168 received LEN alone. The LEN-TACE group had significantly longer mOS and mPFS (17.8 vs. 11.5 months, 10.6 vs. 6.4 months; *P* < 0.001). However, lenvatinib use is associated with some drug-related AEs, including fatigue, anorexia, hypertension, hand-foot syndrome, diarrhea, and hypothyroidism [43]. Studies have reported that fatigue, anorexia, hand-foot syndrome, and diarrhea typically occur within 1 month of starting lenvatinib treatment, while hypertension often occurs by day 4 [44]. Among these common AEs, anorexia is closely associated with treatment efficacy [45]. Additionally, fatigue and anorexia are risk factors for treatment discontinuation [46].Common side effects of TACE include abdominal pain, fever, vomiting, and fatigue [47]. For patients with poor physical condition, undergoing TACE followed by lenvatinib treatment may further deteriorate their physical status, making them unable to receive subsequent tumor-related therapies and potentially affecting their treatment adherence. Chia-Yang Hsu et al. [48] reported that physical condition is an independent prognostic factor for patients with HCC. Therefore, it is crucial to monitor the physical condition of patients.

In this study, we aimed to evaluate the efficacy of combining TACE, lenvatinib, and icaritin in patients with uHCC and compromised performance status. The TACE + LEN+Icaritin group exhibited significantly higher ORR and DCR (66.7% vs. 28.6% and 85.7% vs. 53.6%, respectively; *P* < 0.05), along with a prolonged mPFS (8.6 months vs. 4.1 months; *P* < 0.001). The ORR of 66.7% in the TACE + LEN+Icaritin group is notably higher than rates historically reported for TACE alone or some systemic therapies in advanced HCC. Several factors may contribute to this finding.While the complementary roles of TACE (local tumor debulking) and lenvatinib (anti-angiogenesis) are well-established, the contribution of icaritin warrants hypothesis-driven consideration based on prior research. Preclinical and early-phase clinical studies suggest that icaritin possesses immunomodulatory and direct anti-tumor properties [49, 50]. It is hypothesized that icaritin may remodel the tumor microenvironment, potentially through mechanisms inferred from other studies, such as modulation of inflammatory signaling (e.g., NF-κB) and immune cell activity (e.g., affecting MDSCs or T-cell function) [51]. These proposed mechanisms provide a plausible biological rationale for the observed improvement in patient performance status and may contribute to enhanced tumor control within the combination, although they were not directly measured in this clinical study. Therefore, the improved ECOG scores and lower rates of fatigue/anorexia in the combination group, while encouraging, should be interpreted as a clinically valuable effect of the regimen as a whole, the specific mediators of which remain to be delineated in future biomarker-integrated trials. As reported by Canjing Zhang et al. [52], icaritin influences several pivotal signaling pathways governing cell fate, including CDK-dependent cascades, mitogen-activated protein kinases (MAPKs), AKT serine/threonine kinase, STAT3, and p53. It has demonstrated substantial antitumor activity across multiple malignant cell lines, and its combination with other therapeutic modalities may yield synergistic efficacy [53]. Additionally, Piao Luo et al. [54] revealed that icaritin suppresses the growth, proliferation, and migration of HCC cells through the induction of mitophagy and apoptosis. Intriguingly, inhibition of mitophagy was found to potentiate the pro-apoptotic and anticancer effects of icaritin.

Post-treatment, there were no significant differences between the two groups in terms of total bilirubin, ALT, AST, WBC, RBC, and PLT levels (*P* > 0.05), indicating that the combination of TACE, lenvatinib, and icaritin is safe. Both groups experienced high incidences of abdominal pain, fever, and vomiting as treatment-related AEs, but there were no statistically significant differences (*P* > 0.05). These AEs are primarily associated with post-embolization syndrome following TACE, consistent with findings from other studies [55]. Although the TACE + LEN+Icaritin group had slightly higher incidences of hypertension and diarrhea compared to the TACE group, the differences were not statistically significant (*P* > 0.05). The incidence of hand-foot syndrome (all grades) was higher in the TACE + LEN+Icaritin group than in the TACE group (*P* = 0.004), which is likely due to the use of lenvatinib. However, interestingly, the incidence of fatigue and anorexia (all grades) was lower in the TACE + LEN+Icaritin group compared to the TACE group (*P* < 0.05), with 66.7% of patients in the TACE + LEN+Icaritin group experiencing improvement in physical condition, significantly higher than in the TACE group (*P* = 0.022). After treatment, the proportion of patients with an ECOG score < 2 in the TACE + LEN+Icaritin group was significantly higher than in the TACE group (*P* = 0.035). The results of this study suggest that the combination of TACE, lenvatinib, and icaritin can significantly improve the physical condition of uHCC patients with an ECOG score of 2. This may be due, on one hand, to the higher ORR and DCR in the TACE + LEN+Icaritin group, leading to better tumor control and reduced negative effects of the tumor on the patients, thus improving their physical condition. On the other hand, it may be related to the anti-tumor, immune-modulating, and anti-inflammatory effects of icaritin. Xiaoxia Tang et al. [56] reported that one HCC patient experienced no discomfort after TACE and icariin treatment, with no recurrence of abdominal pain and distension, and a weight gain of 3 kg. Yan Sun et al. [57] reported that in 33 patients with advanced HCC treated with icaritin, the median disease progression time in the icaritin group was nearly twice that of the control group. The incidence of AEs was extremely low, with the overall incidence of grade 3 or higher AEs being less than 10%.

A recognized limitation of this study is its two-group design, which precludes a direct evaluation of the individual contributions of lenvatinib and icaritin to the observed efficacy. The superior outcomes in the triple-therapy group likely arise from a multi-targeted synergistic effect. While preclinical models suggest icaritin can inhibit HCC cell proliferation and synergize with other agents, and clinical data support the efficacy of TACE-lenvatinib combination, the specific incremental benefit of adding icaritin to the TACE-lenvatinib backbone in this patient population remains to be quantified. Future prospective, randomized studies are warranted to dissect the individual and synergistic effects of each component. Second, this study was conducted at a single center with a relatively small sample size, which may limit the statistical power of subgroup analyses and the generalizability of the findings. Third, the assessment of combined status was primarily based on ECOG performance status and standard laboratory tests. Future prospective studies should incorporate serial monitoring of dynamic biomarkers to better capture the comprehensive biological and physiological response to this combination therapy. Additionally, the use of subsequent therapies after documented progression, as mentioned in the methods, represents a potential confounding factor for any future analysis of post-progression. Finally, this study reports PFS as its primary efficacy endpoint. Mature OS data are not yet available, which limits the assessment of the regimen’s ultimate impact on long-term survival. However, PFS remains a validated and clinically meaningful endpoint in HCC, particularly for assessing the early efficacy of novel combinations.

## Conclusion

For patients with uHCC and an ECOG score of 2, the combination of TACE, lenvatinib, and icaritin offers higher ORR and DCR, as well as longer mPFS compared to TACE alone. A higher proportion of patients in the TACE + LEN+Icaritin group showed significant improvement in physical condition. In summary, the results of this study indicate that the combination of TACE, lenvatinib, and icaritin demonstrates good efficacy and safety in treating uHCC patients with poor physical condition, potentially making this combination therapy a viable option for improving prognosis and quality of life in these patients. Furthermore, the results confirm that the combination of TACE, lenvatinib, and icaritin provides significant and consistent PFS benefits across key clinical subgroups. Multivariable modeling establishes this regimen as an independent predictor of improved outcomes, irrespective of tumor burden or liver function.

## Supplementary Information

Supplementary Material 1.

## Data Availability

The datasets used during the current study are available from the corresponding author upon request.

## Abbreviations

TACE: Transarterial chemoembolization
HAIC: Hepatic arterial infusion chemotherapy
uHCC: Unresectable hepatocellular carcinoma
HCC: Hepatocellular carcinoma
CR: Complete Response
PR: Partial Response
SD: Stable Disease
PD: Progressive Disease
ORR: Objective response rate
DCR: Disease control rate
PFS: Progression-free survival
OS: Overall Survival
CT: Computed Tomography
MRI: Magnetic Resonance Imaging
TKIs: Tyrosine kinase inhibitors
AEs: Adverse events
CTCAE: Common Terminology Criteria for Adverse Events
mRECIST: Modified Response Evaluation Criteria in Solid Tumors
ECOG: Eastern Cooperative Oncology Group Performance Status
BCLC: Barcelona Clinic Liver Cancer
WHO: World Health Organization
AFP: Alpha-Fetoprotein
ALT: Alanine Aminotransferase
AST: Aspartate Aminotransferase
WBC: White blood cell
RBC: Red blood cell
PLT: Platelet
HIF-1α: Hypoxia-inducible factor-1α
VEGFR: Vascular Endothelial Growth Factor Receptor
FGFR: Fibroblast Growth Factor Receptor
PDGFR: Platelet-Derived Growth Factor Receptor
TNF-α: Tumor Necrosis Factor-alpha
IL-6: Interleukin-6
PD-L1: Programmed Death-Ligand 1
